# The relationship between illness uncertainty and preoperative anxiety in surgical patients: the moderating role of mental health literacy

**DOI:** 10.3389/fpsyg.2026.1789888

**Published:** 2026-04-24

**Authors:** Xinyu Shi, Wenjing Wang, Liping Du, Yan Liang, Haoran Xu, Haoqing Geng

**Affiliations:** 1 Department Central Operating Room, People's Hospital of Henan University of Chinese Medicine, Zhengzhou, Henan, China; 2 Department of Nursing, People's Hospital of Henan University of Chinese Medicine, Zhengzhou, Henan, China; 3 Department of Surgery System, People's Hospital of Henan University of Chinese Medicine, Zhengzhou, Henan, China

**Keywords:** cross-sectional study, illness uncertainty, mental health literacy, preoperative anxiety, surgical patients

## Abstract

**Objective:**

Preoperative anxiety is a common and clinically significant psychological condition among surgical patients, and illness uncertainty is considered an important psychological stressor for preoperative anxiety. Mental health literacy is an intervenable psychological resource, and its role in preoperative anxiety remains underexplored. This study aims to investigate the relationship between illness uncertainty and preoperative anxiety in surgical patients and further examine the moderating role of mental health literacy in this relationship.

**Methods:**

A cross-sectional study design was adopted. From August to October 2025, 342 surgical patients were conveniently sampled from a tertiary hospital in central China. The Mishel Illness Uncertainty Scale (MUIS), State-Trait Anxiety Inventory–State subscale (STAI-S), and the Short Version of the Mental Health Literacy Questionnaire for Adults (MHLq-SVa) were used for assessment. Descriptive statistics, univariate analysis, and Pearson correlation analysis were conducted. Multivariate linear regression and moderation analysis were used to test the potential moderating effects of mental health literacy in the relationship between illness uncertainty and preoperative anxiety.

**Results:**

Illness uncertainty was significantly positively correlated with preoperative anxiety (*r* = 0.411), and mental health literacy was significantly negatively correlated with preoperative anxiety (*r* = −0.417) and illness uncertainty (*r* = −0.386), all with *P* < 0.001. Moderation analysis showed that mental health literacy significantly moderated the relationship between illness uncertainty and preoperative anxiety (*P* for interaction < 0.001). Among patients with lower mental health literacy (Mean-SD), the association between illness uncertainty and preoperative anxiety was stronger, while among those with higher mental health literacy (Mean + SD), this association significantly attenuated.

**Conclusion:**

Mental health literacy plays a moderating role in the relationship between illness uncertainty and preoperative anxiety in surgical patients. Higher levels of mental health literacy are associated with a weaker association between illness uncertainty and preoperative anxiety, while lower levels are associated with a stronger association.

## 1 Introduction

Anxiety is defined as an emotional response to the anticipated threat in the future or an imminent threat, which can be either real or perceived (First, 2013). Preoperative anxiety is considered a type of state anxiety related to the upcoming surgery, and it is associated with individual personality traits and coping processes (Kassahun et al., 2022). Previous studies have shown that preoperative anxiety is prevalent among surgical patients, with an incidence rate of approximately 11% to 80% (Kassahun et al., 2022; Nigussie et al., 2014; Mavridou et al., 2013). In a survey covering over 16,000 perioperative patients, the worst experience for surgical patients during the perioperative period was anxiety (Walker et al., 2016). Anxiety shows adverse effects on the prognosis of patients, including pain management, patient safety, overall nursing quality and patient satisfaction (Bello et al., 2025; Wang et al., 2022). Timely identification and intervention of preoperative anxiety could reduce the psychological and physiological burdens of patients (Mao et al., 2024; Tulloch and Rubin, 2019).

Illness uncertainty refers to the inability to clearly define things related to the disease (Mishel, 1988). This is a cognitive state accompanied by a sense of ambiguity regarding disease symptoms, diagnosis, treatment, and prognosis (Mishel, 1988, 1990; McCormick, 2002). Michel's theory on illness uncertainty consists of four main components: (a) the generation of uncertainty by the cause, (b) the assessment of uncertainty, (c) coping with uncertainty, and (d) adaptation (Mishel, 1988; Mishel and Braden, 1988). Illness uncertainty is closely associated with a variety of adverse psychological outcomes, including anxiety, depression, emotional distress, and psychological adaptation disorders (Skojec et al., 2025; Cui et al., 2021). The psychological impact of illness uncertainty in groups such as patients with chronic diseases and tumors has been explored to a certain extent (Cheng et al., 2022; Bailey et al., 2021). However, in surgical-related studies, especially during the perioperative period, the relationship between illness uncertainty and preoperative anxiety remains understudied.

Mental health literacy is a concept that was proposed based on health literacy. Initially, Jorm and his colleagues defined it as knowledge and beliefs that help in identifying, coping with, or preventing mental disorders (Jorm et al., 1997). This definition regards mental health literacy as the understanding and mastery of knowledge about mental illnesses and is considered as narrow mental health literacy. Subsequently, some researchers proposed that the academic community should focus on the knowledge and skills that individuals need to maintain their mental health from the perspective of positive mental health (Bjørnsen et al., 2017). Ming and Chen combined their previous viewpoints and, from a broader perspective, defined mental health literacy as the ability to comprehensively apply mental health knowledge, skills and attitudes to maintain and promote mental health (Ming and Chen, 2020). From the perspective of psychological empowerment, mental health literacy is not only a psychological resource but also a capability to self-improve in understanding and managing mental health (Jorm, 2012). Currently, mental health literacy is regarded as a promising intervention target for improving personal mental health (Campos et al., 2022). Mental health literacy is not well-studied in the field of surgery, but it may play an important role considering patients faced with high psychological burden before surgery (Chang et al., 2020). In surgical patients, especially during the perioperative stage, studies on the relationship between mental health literacy and preoperative anxiety are limited.

Although there are many studies on preoperative anxiety, the interplay between illness uncertainty in surgical patients, mental health literacy, and preoperative anxiety remains insufficiently understood. Based on the stress-cognitive appraisal-response theory, we propose that mental health literacy may act as a moderating factor between the stressor (illness uncertainty) and the stress response (preoperative anxiety), influencing how illness uncertainty affects anxiety levels. Moreover, several studies have found that those with lower mental health literacy tend to exhibit higher anxiety (Jessup et al., 2018, 2017; Shahid et al., 2022; Albayrak, 2023), indicating a negative correlation between mental health literacy and anxiety. Based on the application of the stress-cognitive appraisal-response theory model and the review of relevant literature, we hypothesized that mental health literacy plays a moderating role in the relationship between illness uncertainty and surgical anxiety.

Therefore, this study focused on surgical patients as the research subjects and conducted a cross-sectional survey. We adopted the concept model of moderation to incorporate mental health literacy support as a moderator variable, which hypothesized that mental health literacy and illness uncertainty interact with each other and affect the association between illness uncertainty and preoperative anxiety. Our research hypotheses are as follows: (1) Illness uncertainty is negatively correlated with mental health literacy, while illness uncertainty is positively correlated with preoperative anxiety in surgical patients; (2) Mental health literacy is negatively correlated with preoperative anxiety in surgical patients; (3) Mental health literacy plays a moderating role in the positive association between illness uncertainty and preoperative anxiety. We hope to reveal the associations among illness uncertainty, mental health literacy, and preoperative anxiety in surgical patients, and to provide insights for potential methods to address preoperative anxiety related to illness uncertainty in surgical patients.

## 2 Methods

### 2.1 Research design

This study is a cross-sectional study, following the guidelines of the Strengthening the Reporting of Observational Studies in Epidemiology (STROBE) statement: guidelines for reporting observational studies (von Elm et al., 2007).

### 2.2 Design and sampling

Patients with surgery to perform (*N* = 342) were recruited from a tertiary hospital in central China using a convenient sampling method. Data collection was conducted from August to October 2025. Inclusion criteria: Surgical patients 24–48 h before the operation, aged 18 years or older, willing to participate in the study. Those with severe mental disorders/cognitive impairments during data collection; significant difficulties in understanding the items; inability to communicate normally with the researchers; and those unable to complete the questionnaire were excluded. Participants were informed of the research objectives, significance, potential risks, confidentiality protocols, and the voluntary nature of participation. Written documentation of informed consent was obtained via the questionnaire after participants agreed to take part in the study. To mitigate common method bias, respondents were assured of the anonymity of their responses, which helped reduce social desirability bias and encouraged candid feedback. Subsequently, participants completed the questionnaire within 10–15 min and returned it to the data collection personnel.

### 2.3 Assessments

#### 2.3.1 Sample characteristics

Participants completed questionnaires regarding demographic characteristics and factors potentially related to anxiety, including gender, age, educational level, type of residence, disease type, whether it was the first surgery, and sleep quality at night.

#### 2.3.2 Measurement of preoperative anxiety

The assessment tool used was the State-Trait Anxiety Inventory–State (STAI-S), It is State-Trait Anxiety Inventory (STAI) one of the two sub-scales and can be used independently (Spielberger et al., 1970). STAI first edition (STAI-Form X) was published in 1970. In 1979, Spielberger and his colleagues revised the STAI-Form X, and the revised version (STAI-Form Y) was introduced in 1980, was later translated into Chinese in 1988 (Wang et al., 1999). STAI-S is used to evaluate the intensity of each symptom of the individual at the moment. It contains 20 items, each rated on a scale of 1 to 4, where 1 indicates “absolutely not,” 2 indicates “somewhat,” 3 indicates “moderate,” and 4 indicates “very obvious.” Ten of the 20 items are reverse scored. The total score of the scale ranges from 20 to 80, with higher scores indicating a more severe level of anxiety. The overall Cronbach's α coefficient of the STAI-S in this study was 0.94.

#### 2.3.3 Measurement of illness uncertainty

The MUIS was developed by Mishel in 1981 and consists of two dimensions: ambiguity and unpredictability, with 30 items (Mishel, 1981). In 2018, Ye et al. translated and localized it into Chinese and compiled 20 items, including the following three dimensions: ambiguity, lack of clarification, and unpredictability (Ye et al., 2018). This scale uses the Likert five-point rating method, with a total score ranging from 20 to 100. The higher the score, the higher the level of patients' illness uncertainty. The overall Cronbach's α coefficient of MUIS in this study was 0.93.

#### 2.3.4 Measurement of mental health literacy

The Adult Mental Health Literacy Questionnaire Short Version (MHLq-SVa) was developed by (Campos et al. 2022), and translated and culturally adapted into Chinese by (Su et al. 2024). The MHLq-SVa questionnaire consists of 16 items and is divided into four dimensions: (1) Understanding of mental health issues; (2) Incorrect beliefs/ stereotypes; (3) Skills for seeking help and emergency treatment and (4) self-help strategies. This scale uses the Likert five-point scale (1 = “completely disagree,” 5 = “completely agree”), and the higher the total score, the higher the individual's mental health literacy level. The overall Cronbach's α coefficient of the MHLq-SVa in this study was 0.93.

### 2.4 Data analysis

Descriptive statistics were conducted for all participants. Categorical data were reported in terms of frequency and percentage. The average and standard deviation of illness uncertainty, anxiety, and mental health literacy were calculated for different categories. Two sample *t*-tests or one-way ANOVA were used to compare groups based on the number of categories, and the differences of the three variables at the single-factor level were explored. Pearson correlation analysis was used to investigate the correlation among illness uncertainty, anxiety, and the total score of mental health literacy. Then, an exploratory factor analysis was conducted on all the scale items using the Harman single-factor test. The results showed that the first unrotated factor explained 27.4% of the total variance, which was below the critical value of 50%, indicating that the common method bias in this study was not severe. The VIF of each variable was calculated, and a VIF < 5 was defined as the absence of significant multicollinearity, all VIF values were below 2.5, indicating no serious multicollinearity concerns (Supplementary Table 1). Breusch-Pagan test was conducted to assess heteroscedasticity in the regression model. Residuals were tested for linearity using the Residuals vs. Fitted plot, normality using the Q-Q Residuals plot, homoscedasticity using the Scale-Location plot, and outliers/high leverage points using the Residuals vs. Leverage plot (Supplementary Figure 1). Multiple linear regression was then used to explore the relationship between the significant variables at the univariate level and preoperative anxiety, while including illness uncertainty and mental health literacy. The moderating effect of mental health literacy on the relationship between illness uncertainty and preoperative anxiety was tested using the “lavaan” package in R. The interaction *P*-value was calculated, and the association between illness uncertainty and preoperative anxiety at three levels of mental health literacy (Mean-SD, Mean, and Mean + SD) was also examined. For moderation analysis, illness uncertainty and mental health literacy were mean-centered prior to creating the interaction term to reduce multicollinearity. The interaction term was computed as the product of the centered variables. Assumptions of linearity, homoscedasticity, and normality of residuals were checked and met. A *post-hoc* sensitivity analysis indicated that with a sample size of 342, a two-tailed α = 0.05, and power = 0.80, the minimum detectable effect size for moderation (*f*^2^) was 0.023, suggesting adequate power to detect small interaction effects. Covariates for all multivariable models (both multivariate linear regression and moderation analysis) were selected based on theoretical relevance from prior literature and univariate associations (*p* < 0.05). The following covariates were included in all adjusted models: age, education level, surgical department, whether the first operation, and nighttime sleep quality. This harmonized approach ensures comparability across analyses. All statistical analyses were completed using R (version 4.5.0), with a two-tailed *P*-value < 0.05 considered statistically significant. Bootstrap resampling with 5,000 samples was applied, and 95% bias-corrected bootstrap confidence intervals (CIs) were used to estimate the interaction effects.

## 3 Results

### 3.1 Participant characteristics

A total of 342 patients who underwent surgery within 48–24 h before the study were included. Among them, 125 were male (36.5%) and 217 were female (63.5%). The age distribution was as follows: 116 cases (33.9%) were aged 18–39 years, 139 cases (40.6%) were aged 40–59 years, and 87 cases (25.4%) were aged 60 years or above. There were 114 cases (33.3%) in general surgery, 60 cases (17.5%) in orthopedics, 88 cases (25.7%) in gynecology and obstetrics, 6 cases (1.8%) in thoracic surgery, 41 cases (12.0%) in urology, and 33 cases (9.6%) in Ophthalmology; 148 cases (43.3%) were first-time surgery patients, with details shown in Table 1.

**Table 1:** Univariate analysis (*N* = 342).

Variable	Category	Number (%)	Illness uncertainty			Preoperative anxiety			Mental health literacy		
			**Mean** ±**SD**	***t*** **/** ***F***	***P***	**Mean** ±**SD**	***t*** **/** ***F***	***P***	**Mean** ±**SD**	***t*** **/** ***F***	***P***
Gender	Male	125 (36.5%)	43.6 ± 1732	0.68	0.495	36.1 ± 11.9	−2.09	0.037	68.1 ± 9.41	0.29	0.771
	Female	217 (63.5%)	42.3 ± 16.7			38.8 ± 11.1			67.8 ± 10.3		
Age	18–39 years	116 (33.9%)	38.2 ± 15.3	6.826	0.002	37.6 ± 10.8	0.07	0.932	69.6 ± 9.27	3.24	0.044
	40–59 years	139 (c40.6%)	44.7 ± 15.7			38.1 ± 11.7			67.6 ± 9.97		
	60 years or above	87 (25.4%)	45.9 ± 19.2			37.7 ± 12.1			66.1 ± 10.6		
Educational level	Primary school or below	50 (14.6%)	49.0 ± 18.1	4.967	< 0.001	40.0 ± 11.1	0.758	0.58	66.3 ± 11.7	1.44	0.211
	Junior high school	80 (23.4%)	44.4 ± 16.8			37.4 ± 11.9			67.3 ± 10.5		
	High school	71 (20.8%)	45.8 ± 18.6			38.7 ± 11.9			67.7 ± 9.10		
	College	68 (19.9%)	40.6 ± 15.5			36.3 ± 11.2			67.9 ± 9.78		
	Bachelor's degree	60 (17.5%)	36.3 ± 12.8			37.0 ± 11.4			68.8 ± 9.58		
	Master's degree or above	13 (3.8%)	34.1 ± 12.1			38.5 ± 10.7			74.2 ± 4.32		
Residence	Urban	240 (70.2%)	41.9 ± 16.6	−1.539	0.125	37.3 ± 11.5	−1.338	0.185	68.0 ± 9.79	0.29	0.769
	Rural	102 (29.8%)	45.0 ± 17.2			39.1 ± 11.5			67.6 ± 10.5		
Surgical department	General surgery	114(33.3%)	42.1 ± 16.2	1.709	0.132	38.6 ± 12.0	2.41	0.036	66.7 ± 11.2	1.25	0.28
	Orthopedics	60(17.5%)	45.2 ± 19.7			36.5 ± 11.4			70.0 ± 7.45		
	Gynecology and obstetrics	88(25.7%)	40.6 ± 14.7			39.9 ± 10.2			67.6 ± 10.5		
	Thoracic surgery	6(1.8%)	41.7 ± 16.0			39.5 ± 12.5			68.8 ± 10.7		
	Urology	41(12.0%)	48.4 ± 17.6			37.1 ± 11.9			66.6 ± 10.3		
	Ophthalmology	33(9.6%)	40.0 ± 16.9			32.4 ± 11.0			69.7 ± 7.15		
Whether the first operation	The first operation	148 (43.3%)	42.9 ± 15.9	0.111	0.911	39.3 ± 11.7	2.072	0.036	68.3 ± 9.21	0.68	0.497
	Not the first operation	194 (56.7%)	42.7 ± 17.6			36.7 ± 11.2			67.6 ± 10.5		
Nighttime sleep quality	Very poor	14 (4.1%)	48.7 ± 17.8	3.582	0.007	41.6 ± 11.5	11.52	< 0.001	70.4 ± 8.77	0.96	0.423
	Poor	18 (5.3%)	51.9 ± 16.1			47.3 ± 9.63			66.4 ± 8.89		
	Average	92 (26.9%)	44.4 ± 14.5			41.9 ± 9.29			66.9 ± 8.65		
	Good	75 (21.9%)	38.0 ± 14.7			36.7 ± 10.4			67.2 ± 10.3		
	Excellent	143 (41.8%)	42.5 ± 18.3			34.2 ± 12.0			68.8 ± 10.8		

The thoracic surgery subgroup contained only 6 patients (1.8%); findings related to this subgroup should be interpreted with particular caution due to the small sample size.

### 3.2 Univariate analysis

In this study, female patients had significantly higher preoperative anxiety scores compared to male patients (38.8 ± 11.1 vs. 36.1 ± 11.9; *P* = 0.037). Significant differences in preoperative anxiety were also observed across surgical departments, with higher anxiety scores in thoracic surgery and gynecology patients (*P* < 0.05), and in patients undergoing their first surgery (*P* < 0.05). Regarding illness uncertainty, significant differences were found between age groups (*P* = 0.002), with patients aged 60 and above scoring the highest (45.9 ± 19.2). Uncertainty of illness also varied significantly by education level, with the highest scores in patients with primary education or below (*P* < 0.001). Poorer sleep quality was associated with higher illness uncertainty (*P* < 0.05). As for mental health literacy, significant differences were found by age group (*P* = 0.044), with the highest scores in patients aged 18–39 (69.6 ± 9.27), as shown in Table 1. Univariate analyses were conducted to explore potential associations and should be interpreted as exploratory. No correction for multiple comparisons was applied, as these analyses were intended to inform covariate selection for multivariable models.

### 3.3 Pearson correlation analysis

The mean and standard deviation of the overall scores of the three variables are as follows: illness uncertainty: 42.80 ± 16.85; preoperative anxiety: 37.80 ± 11.48; mental health literacy: 67.81 ± 10.00. Illness uncertainty was significantly positively correlated with preoperative anxiety (*r* = 0.4113); mental health literacy was significantly negatively correlated with preoperative anxiety (*r* = −0.4166); mental health literacy was significantly negatively correlated with illness uncertainty (*r* = −0.3864), and all *P* values were < 0.001, as shown in Table 2.

**Table 2:** Pearson correlation analysis.

Variable	Mean ±SD	MUIS	STAI-S	MHLq-SVa
MUIS	42.80 ± 16.85	1		
STAI-S	37.80 ± 11.48	0.4113	1	
MHLq-SVa	67.81 ± 10.00	−0.3864	−0.4166	1

Note: All *P* values were < 0.001.

### 3.4 Multivariate linear regression analysis

After including the significant features from the univariate analysis and the illness uncertainty, as well as mental health literacy, in the multivariate linear regression analysis, non-first surgery (β = −3.033, *P* = 0.004), poor sleep quality (β = −6.853, *P* = 0.010), illness uncertainty (β = 0.185, *P* < 0.001), and mental health literacy (β = −0.332, *P* < 0.001) were all significant influencing factors of preoperative anxiety. This model could explain 33.9% of the variance (adjusted *R*^2^ = 0.339), as shown in Table 3.

**Table 3:** Multivariate linear regression analysis.

Variable	*B*	95% CI	SE	*t*	*p*	*R* ^2^	*F*	*P*
						**(Adj**.*R*^2^**)**		
						0.364	14.43	< 0.001
Gender [2]	1.603	[−0.807, 4.013]	1.229	1.305	0.192	(0.339)		
Surgical department [2]	−0.921	[−3.942, 2.100]	1.540	−0.598	0.550			
Surgical department [3]	1.057	[−1.702, 3.816]	1.408	0.751	0.453			
Surgical department [4]	1.591	[−6.118, 9.300]	3.934	0.404	0.686			
Surgical department [5]	−0.324	[−3.869, 3.221]	1.809	−0.179	0.858			
Surgical department [6]	−2.864	[−6.577, 0.849]	1.895	−1.511	0.132			
Whether the first operation [1]	−3.033	[−5.088, −0.978]	1.048	−2.893	0.004			
Nighttime sleep quality [2]	2.898	[−3.672, 9.468]	3.356	0.863	0.359			
Nighttime sleep quality [3]	0.782	[−6.098, 4.534]	2.712	−0.288	0.773			
Nighttime sleep quality [4]	−4.115	[−9.574, 1.344]	2.785	−1.478	0.140			
Nighttime sleep quality [5]	−6.853	[−12.054, −1.652]	2.654	−2.582	0.010			
Total score of illness uncertainty	0.185	[0.118, 0.252]	0.034	5.411	< 0.001			
Total score of mental health literacy	−0.332	[−0.442, −0.222]	0.056	−5.900	< 0.001			

Gender, 1 = Male (Reference), 2 = Female; Surgical department, 1 = General surgery (Reference), 2 = Orthopedics, 3 = Gynecology and obstetrics, 4 = Thoracic surgery, 5 = Urology, 6 = Ophthalmology; Whether the first operation, 0= The first operation (Reference), 1 = Not the first operation; Nighttime sleep quality, 1 = Very poor (Reference), 2 = Poor, 3 = Average, 4 = Good, 5 = Excellent.

### 3.5 Moderation analysis

**Figure 1:**
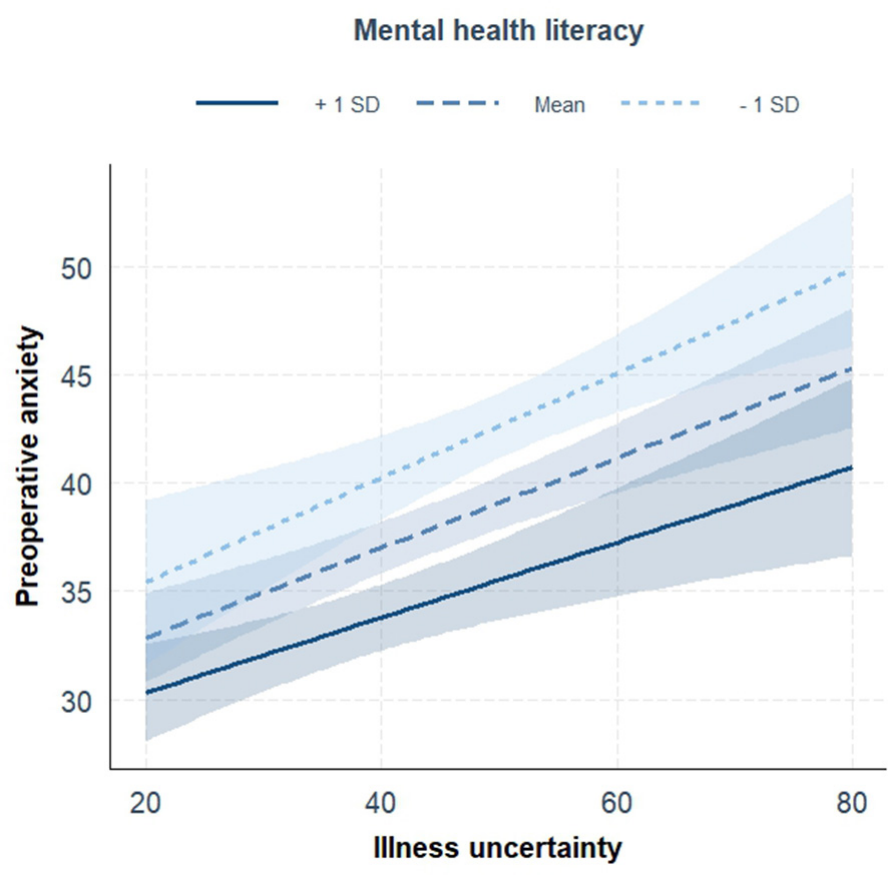
The moderating role of mental health literacy in the relationship between illness uncertainty and preoperative anxiety.

After controlling for the same set of covariates used in the multivariate regression analysis (age, education level, surgical department, whether the first operation, and nighttime sleep quality), the interaction term between illness uncertainty and mental health literacy significantly predicted preoperative anxiety (*P* for interaction < 0.001). The interaction term added a small but statistically significant increment in explained variance (Δ*R*^2^ = 0.028, *p* < 0.001), indicating that the moderation effect, while statistically detectable, is modest in magnitude. When mental health literacy was at a low level (Mean – SD), the predictive effect of illness uncertainty on preoperative anxiety was the strongest (β = 0.21, *P* < 0.001); when mental health literacy was at a medium level (Mean), this predictive effect was still significant (β = 0.21, *P* < 0.001); when mental health literacy was at a high level (Mean + SD), this predictive effect significantly weakened (β = 0.16, *P* < 0.001), as shown in Table 4 and Figure 1.

**Table 4:** Relationship between illness uncertainty and preoperative anxiety at different levels of mental health literacy.

Term	β	95% CI	SE	*t*-value	*p*
16-7.4,-39242pt**Interaction term** (illness uncertainty × MHL)	0.009	[0.004, 0.014]	0.003	3.82	< 0.001
Simple slopes					
MHL at Mean – SD (low)	0.21	[0.10, 0.32]	0.05	3.88	< 0.001
MHL at Mean (moderate)	0.21	[0.14, 0.28]	0.04	5.82	< 0.001
MHL at Mean + SD (high)	0.16	[0.07, 0.25]	0.05	3.71	< 0.001

ΔR^2^ attributable to the interaction term = 0.028 (p < 0.001). Simple slopes were estimated using 5,000 bootstrap resamples. Unstandardized coefficients (B) are reported. The small magnitude of the interaction coefficient (0.009) reflects the scaling of the product term and should be interpreted alongside the ΔR^2^.

## 4 Discussion

To our knowledge, this study is among the first to simultaneously introduce illness uncertainty and mental health literacy in surgical patients and conduct a systematic analysis using the moderation model, providing a new perspective for understanding the psychological correlates of preoperative anxiety. Our main findings showed that higher levels of illness uncertainty were associated with higher levels of preoperative anxiety. Additionally, mental health literacy was significantly negatively correlated with preoperative anxiety and significantly negatively correlated with illness uncertainty. Further analysis revealed that mental health literacy played a moderating role in the relationship between illness uncertainty and preoperative anxiety. These results suggest that mental health literacy may be one of the important psychological constructs that may be targeted to lower the detrimental impact of illness uncertainty on preoperative anxiety.

For clinical context, previous studies have suggested that STAI-S scores ≥ 40 indicate clinically significant state anxiety in surgical populations (Skapinakis, 2014; Emons et al., 2019). In our sample, approximately half of patients scored above this threshold, indicating that a substantial proportion experienced clinically meaningful preoperative anxiety. In the univariate analysis, this study found that female patients, first-time surgery patients, patients from certain surgical departments (such as thoracic surgery and obstetrics and gynecology), and those with poor sleep quality exhibited higher levels of preoperative anxiety. These findings are consistent with previous studies, such as a study in Northeast China involving 997 preoperative patients, where being female, undergoing high-risk surgeries, and experiencing insomnia were associated with higher preoperative anxiety levels (Li et al., 2021). A cross-sectional study in Germany, involving over 3,087 adult patients undergoing elective surgery, also identified that being female as a factor associated with preoperative anxiety (Eberhart et al., 2020). Based on the analysis of biological factors and psychosocial factors: the biological factor study found that potential influences of brain structure, genetic factors, and hormonal fluctuations were the reasons for the exacerbation of anxiety in women; in the psychosocial factors, masculinity might be a protective factor for anxiety development, while femininity might be a risk factor (Farhane-Medina et al., 2022). Throughout the social process, women are more likely to seek help; they are allowed and encouraged to express fear and worry, and are more often inclined to rely, fear, be passive and obedient (Richmond et al., 2015). In contrast, men are more likely to be taught to control fear and insecurity, and are more often prepared for actions, problem-solving, goal achievement and success (Berke et al., 2018).

Furthermore, for surgical patients who undergo the operation for the first time, due to the lack of previous experience, they have more uncertainties regarding the surgical process, anesthesia risks, and postoperative recovery, which may contribute to higher anxiety levels. Compared to the first surgery, patients reported lower levels of anxiety during the second surgery (Yu et al., 2016; Nijkamp et al., 2004). Meanwhile, differences in preoperative anxiety levels among patients in different surgical departments are perhaps explained by the severity of the disease, the complexity of the surgery, and the patients' subjective perception of the prognosis (Li et al., 2021). Further, the results of this study show that the level of illness uncertainty varies significantly among different age groups, educational levels, and sleep quality groups. The studies by Chen et al. and Li et al. suggest that patients who are older, have a lower educational level, and have poorer sleep quality tend to report higher levels of illness uncertainty (Li et al., 2020; Chen et al., 2022), which are consistent with our study.

Meanwhile, there are differences in mental health literacy among different age groups. Younger patients tend to have higher mental health literacy levels, while older patients tend to have lower levels. Previous studies have suggested that women generally report higher mental health literacy levels than men (Hadjimina and Furnham, 2017; Tay et al., 2018), and younger individuals tend to perform better than older individuals (Hadjimina and Furnham, 2017). However, in this study, there was no significant difference in gender groups, which might be due to differences in assessment tools or cultural differences in different regions.

Notably, we found a significant positive correlation between illness uncertainty and preoperative anxiety. That is, higher levels of illness uncertainty are associated with more severe preoperative anxiety in patients. This finding is consistent with the research results of (Obispo-Portero et al. 2022) and (Cruz-Castellanos et al. 2024). Previous studies have mainly focused on patients with chronic diseases, cancer, and epidemics (Guan et al., 2025; Liu et al., 2025; Giammanco et al., 2015; Zhang, 2017; Dong et al., 2022), while in the special context of the perioperative period, surgical patients, due to the different types of surgeries and invasive operation methods (Friedrich et al., 2022), will further amplify the impact of illness uncertainty on anxiety. The study of illness uncertainty in the perioperative context is relatively rare, and the results of this study to some extent make up for the lack of research in this direction in the surgical field.

Another important finding of this study is that mental health literacy shows a buffering pattern in the positive relationship between illness uncertainty and preoperative anxiety. The moderation analysis results show that in patients with lower levels of mental health literacy, the association between illness uncertainty and preoperative anxiety is stronger; while in patients with higher levels of mental health literacy, this association is significantly weaker. This study's findings regarding the impact of mental health literacy are consistent with previous research (Elyamani et al., 2021; Goodfellow et al., 2023; Spiker and Hammer, 2019). This suggests that mental health literacy, as a “buffer factor,” may reduce the negative emotional responses associated with illness uncertainty. It should be noted, however, that the magnitude of the moderation effect was modest (Δ*R*^2^ = 0.028), suggesting that while mental health literacy significantly moderates the relationship between illness uncertainty and preoperative anxiety, other unmeasured factors likely contribute substantially to the variance in anxiety levels. This modest effect size is consistent with the complexity of preoperative anxiety, which is influenced by multiple psychological, clinical, and demographic factors.

The results of this study suggest potential directions for perioperative psychological care practice. Based on previous research findings and the discoveries of this study, we propose the following hypotheses for future research: during preoperative assessment, special attention may be associated with the anxiety states of female patients, first-time surgery patients, and patients in high-risk departments. Targeted psychological support and intervention measures could be beneficial. In clinical practice, the assessment of illness uncertainty may be important. Healthcare providers can proactively provide detailed and comprehensive treatment plans, prognosis, and family self-care information (Chen et al., 2022). Clear communication and effective doctor-patient interaction may help patients reduce their vague understanding of the disease and surgery. Secondly, mental health literacy, as an interventional psychological resource (Soria-Martínez et al., 2024), from the perspective of health empowerment, may be improved through mental health education, encouraging, empowering, empowering and supporting patients to be responsible for their own disease management, and improving their autonomy in decision-making (Pulvirenti et al., 2014). This could improve patients' ability to recognize and cope with anxiety and may reduce the negative impact of illness uncertainty on preoperative anxiety. Therefore, integrating the enhancement of mental health literacy into the comprehensive perioperative management strategy could be a feasible approach to supporting the preoperative psychological state of patients and facilitating their postoperative recovery. Notably, these suggestions should be interpreted as hypothesis-generating rather than practice-changing, as the current cross-sectional design cannot establish causality and the effect size was modest. Future intervention studies are needed to determine whether enhancing mental health literacy has clinical utility in reducing preoperative anxiety.

The advantage of this study lies in that it is the first to systematically explore the relationship among illness uncertainty, mental health literacy, and preoperative anxiety in surgical patients, and simultaneously examines the associations between these factors using a moderating model. This adds to the understanding of the psychological factors involved in preoperative anxiety. However, this study still has several important limitations. Firstly, this study adopts a cross-sectional design, which cannot directly infer the causal relationship between variables. Future research can further verify the causal path through longitudinal or intervention studies. Secondly, the research subjects are only from a Chinese tertiary hospital, and the sample representativeness is limited. Subsequent research can conduct multi-center studies in different regions and different levels of medical institutions. Third, and most critically, this study did not measure several potentially important confounders that are central determinants of preoperative anxiety, including trait/personality anxiety, history of mental illness, pain conditions, and type of anesthesia. Additionally, surgical severity was only approximated by “surgical department,” which is a coarse proxy; more granular indicators such as elective versus urgent surgery, cancer versus benign indication, and anticipated pain burden were not collected. The absence of these variables significantly limits causal interpretation, and the moderation finding should be interpreted with caution. Fourth, this study collects data through self-assessment questionnaires, which may be affected by social expectation bias. All variables were assessed using self-report questionnaires at a single time point, which may introduce common method bias. Although procedural remedies such as anonymity and scale separation were used, and Harman's single-factor test indicated that common method bias is not a major concern (first factor explained 27.4% of variance, below the 50% threshold), we cannot rule out the possibility of shared method variance. Future research should include objective measures or interviewer-based assessments to obtain more robust results. Finally, future research should consider including trait anxiety, psychiatric history, pain status, anesthesia type, and detailed surgical severity indicators as covariates to improve the accuracy of findings.

## 5 Conclusion

This study indicates that illness uncertainty is associated with higher levels of preoperative anxiety in surgical patients, and mental health literacy shows a moderating pattern in this relationship. Future longitudinal or intervention studies are needed to determine whether enhancing mental health literacy or reducing illness uncertainty can reduce preoperative anxiety levels.

## Data Availability

The raw data supporting the conclusions of this article will be made available by the authors, without undue reservation.
