# Supplementary material for: The relationship between illness uncertainty and preoperative anxiety in surgical patients: the moderating role of mental health literacy
Source: Front Psychol. 2026 Apr 24;17:1789888. doi: 10.3389/fpsyg.2026.1789888 (PMC13153077; doi:10.3389/fpsyg.2026.1789888)

| **Supplementary Table 1 Results of multicollinearity test.** | |
| --- | --- |
| **Variables** | **VIF** |
| MUIS | 1.243 |
| MHLq-SVa | 1.206 |
| Gender | 1.038 |
| Whether the first operation | 1.027 |
| Nighttime sleep quality | 1.109 |

**Supplementary Figure 1 Tests of residuals, normality, homoscedasticity and outliers.**


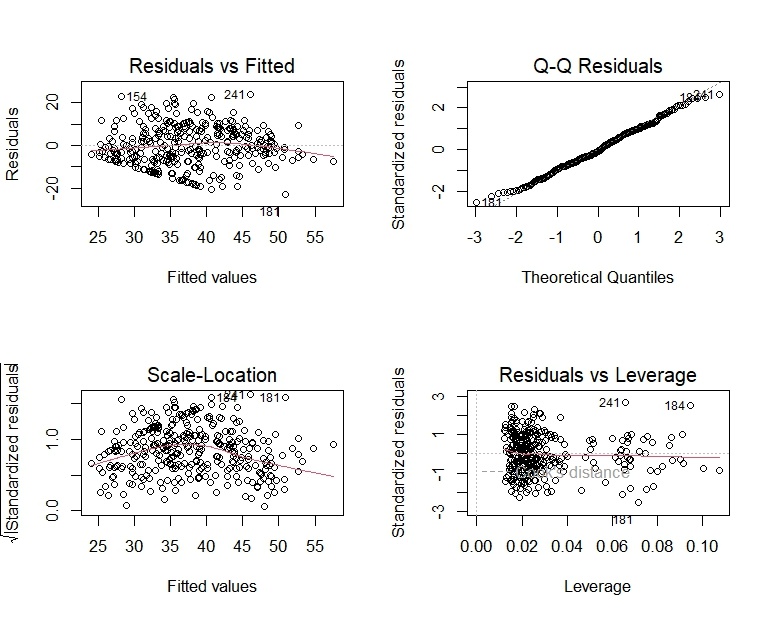

Supplement: Supplementary file 1 [file Table_1.docx]
